# New Paradigms in Nephrology Nursing

**DOI:** 10.7759/cureus.81487

**Published:** 2025-03-31

**Authors:** Andrea Pinto

**Affiliations:** 1 Nursing, Hospital Dr. Nélio Mendonça, Serviço de Saúde da Região Autónoma da Madeira (SESARAM) Entidade Pública Empresarial Regional Autónoma da Madeira (EPERAM), Funchal, PRT

**Keywords:** cardio-renal syndrome, chronic kidney disease (ckd), conservative kidney management, frail patients, nursing care

## Abstract

With the aging of the population and better healthcare, more elderly and frail patients are reaching the terminal stage of chronic kidney disease (CKD). In Portugal, one of the European leaders in the incidence of renal function replacement therapy, hemodialysis and peritoneal dialysis continue to be the focus of treatment, even in patients with multiple comorbidities, low functional reserve, and care dependency. However, several studies have revealed that these therapies in fragile patients do not provide quality of life or a greater survival rate but are associated with increased suffering due to the loss of functional capacity, with a subsequent need to abandon dialysis.

Nephrology nursing in Portugal has always followed this more technical aspect necessary for dialysis. However, it has not dedicated itself to conservative treatment or other nephrology areas requiring differentiated palliative care, such as cardiorenal syndrome (CRS). Conservative treatment is a valid alternative that corresponds to the continuation of comprehensive therapy for patients with CKD without resorting to dialysis techniques. It seeks to prevent the deterioration of residual renal function while alleviating symptoms and complications resulting from disease progression, with personalized palliative care support aimed at optimizing the physical, emotional, and spiritual well-being of the patient and their family.

On the other hand, despite being palliative, patients with CRS can benefit from more invasive techniques such as assisted peritoneal ultrafiltration to control symptoms, along with an educational approach that promotes their empowerment. This need for a paradigm shift led us to implement a specific nursing consultation for conservative treatment and CRS, ensuring personalized monitoring of these illnesses. Palliative care must be an integral part of healthcare, not only for incurable and terminal diseases but also for chronic, advanced, and evolving conditions.

## Introduction and background

The improvement in quality of life and the healthcare services provided to the population have increased average life expectancy and, consequently, the progressive aging of the population. As a result of this process, a clear shift in the epidemiological transition phenomenon emerges, replacing infectious diseases with chronic non-communicable diseases [1,2].

For this reason, the World Health Organization [3] has urged countries to develop appropriate strategies to address chronic conditions, outlining actions to strengthen palliative care as an integral part of treating chronic diseases throughout life. Palliative care has expanded its focus to individuals not only with incurable diseases but also those with prolonged, progressive, and complex conditions. Its primary aim is to prevent suffering and ensure patients' best possible quality of life by alleviating symptoms, facilitating adaptation to the illness, and providing support to their families and caregivers, reducing their burden and assisting them through the grieving process [3,4].

Chronic kidney disease (CKD) falls within this category of prolonged and complex diseases that benefit from a palliative approach, as it is a progressive and inevitably fatal condition unless treated with one of the available renal replacement therapies (RRTs): dialysis or kidney transplantation. However, these treatments do not constitute a cure for the disease [5].

From this perspective, we are witnessing an increasingly aging population with multiple comorbidities and, consequently, growing demands for care. This is due not only to the aging population in Portugal but also to modern lifestyles, which are more sedentary and less healthy. These factors have led to a rise in the prevalence of hypertension, diabetes mellitus, and obesity, which are key contributors to the development of CKD [6].

CKD has thus become one of the major public health challenges in Portugal over the past decades, with its overall prevalence showing an increasing trend, resulting in a rise in the number of patients undergoing RRT. In 2024, the total incidence of new patients starting RRT was 271.6 per million population (pmp). Despite the growing number of patients with multiple comorbidities, vulnerability, and increased care dependency, the incidence of conservative medical treatment remained low, at just 1.7 pmp. Notably, 31% of new patients starting hemodialysis were over 80 years old [7].

Nephrology nursing in Portugal has always aligned with the highly technical approach required for RRTs (hemodialysis and peritoneal dialysis). In fact, prevailing concepts, such as those proposed by Trentini and Cubas [8], define nephrology nursing as a form of palliative care, mediated by cutting-edge technologies within dialysis and hemodialysis units [8].

These concepts do not cover conservative treatment, nor other areas of nephrology that require differentiated palliative care, such as cardiorenal syndrome (CRS). However, in our view, the nurse's role in the area of nephrology is much broader and requires a mastery of knowledge and skills that determine their actions, which range from disease prevention to its treatment.

This includes the implementation of health education programs aimed at disease prevention; specialized nursing consultations according to the stage of kidney disease, with teachings directed not only to the promotion of health and delay of the progression of kidney disease but also to the control of the pathology and associated symptoms; the performance of renal replacement techniques such as hemodialysis and peritoneal dialysis; providing care in the pre- and post-transplant periods and conservative medical treatment; and new areas of intervention in nephrology, such as CRS [9].

## Review

In Portugal, the scope of nurses' work in the field of nephrology has been a topic of debate in order to discuss the need to recognize the nephrology nursing specialty as an autonomous area of practice, based on its extensive knowledge, work throughout the life cycle, exercise in all contexts of care provision, and response to the growing needs of the population with kidney disease, whether isolated or accompanied by multiple pathologies.

Nurses working in this area must understand the difficulty of living with a chronic condition that requires constant care and monitoring. In fact, most patients with kidney disease experience a large number of care transitions related to the natural course of kidney disease until they reach the end stage. The care goals of patients with CKD tend to shift to focus almost exclusively on quality of life rather than survival, with a strong emphasis on emotional, social, and family support [9].

In this final stage of the disease, the support provided to patients is of particular importance, as they face the fear and anguish of difficult decisions that will have repercussions for themselves and their family environment. Thus, they must cope with many physical and psychological stressors to achieve an acceptable quality of life. It is important to note that the family also requires support from healthcare professionals, as they also have their own expectations regarding their lives and the illness [9].

In addition to effective communication, the nurse should promote empowerment strategies to facilitate the patient and their family's adaptation process, thereby facilitating decision-making.

This decision-making process has become increasingly complex. In fact, with socioeconomic and technological development, better living conditions, and greater access to healthcare over the past few decades, there has been a turning point in the treatment of the disease in more severe situations, with increasingly differentiated responses and care, which allow patients to survive longer but also reach a state of greater fragility [10].

Frailty is a multifactorial clinical syndrome influenced by complex and dynamic biological, psychological, cognitive, and social interactions, which can reduce the autonomy and independence of the individual as a result of the loss of functional reserve that is related to age but which can also be associated with the presence of chronic diseases. Frailty is, therefore, a long-term condition that, despite being common in older people, is not an inevitability of aging and can occur in chronic diseases, as is the case with CKD [10].

Nephrology nursing, therefore, plays a crucial role in the care of these patients, especially with increased emphasis on conservative medical management and the development of specialized programs for managing CRS, both of which necessitate holistic and comprehensive care.

Nurse's role in cardiorenal syndrome

CRS is defined as the pathophysiological condition of coexisting cardiac and renal dysfunction [11]. In this syndrome, the heart's abnormal functioning affects the kidneys' abnormal performance, and vice versa. The coexistence of failure of the two organs is usual; that is, renal dysfunction frequently accompanies cardiac failure, and cardiac dysfunction frequently accompanies renal failure; this association quickly leads to a vicious cycle [12].

Epidemiological studies have shown that 25% to 63% of patients with heart disease have some type of CRS [13]. In fact, the incidence of cardiorenal patients doubles in cardiovascular patients over 66 years of age (64.5%), which is associated with a worse prognosis and significantly decreases their survival [14]. The existence of renal injury in the context of heart failure or vice versa is thus a factor of poor prognosis, so early diagnosis and therapeutic intervention are fundamental to improving the survival and quality of life of patients with CRS [15].

In fact, patients with CRS suffer from multiple physical and psychological symptoms that affect their well-being and share experiences that impact all spheres of life [16]. In an attempt to prevent complications and increase survival, patients undergo a very complex therapeutic regimen [14]. However, adherence to this regimen remains low, and traditional educational interventions to improve it have shown limited effectiveness [14,17].

Given this scenario, the World Health Organization [3] and other authors [14,18,19] emphasize the need for healthcare professionals to promote innovative care, combining educational approaches that foster patient empowerment. These measures will improve health outcomes, increase patient satisfaction and quality of life, reduce hospital readmissions, and consequently lower healthcare costs.

Another challenge of CRS is volume overload, which is a common clinical sign and one of the main therapeutic targets. The challenge ranges from individualizing treatment according to the patient's volume status, considering their clinical condition, medical history, risk profile, and comorbidities, to removing excess fluids [20].

However, assessing venous congestion remains one of the greatest clinical challenges today. The nephrology nurse must be able to participate in evaluating patients' hydration status, particularly through the use of the "5Bs approach" proposed by Ronco et al. [21]. The parameters included are balance of fluids (reflected by body weight), blood pressure, biomarkers, bioimpedance vector analysis, and blood volume [21,22].

To optimize and improve the monitoring of venous congestion, the nurse can actively participate in the ultrasonographic assessment of the lung and the inferior vena cava [23] and the determination of the venous excess ultrasound score. Several studies have demonstrated the importance of ultrasonographic assessment in identifying clinically significant systemic venous congestion, allowing for individualized treatment and better patient outcomes [23,24].

The therapeutic strategy should remove excess intravascular and extravascular fluid without causing exacerbation of neurohormonal activation and/or worsening renal function. Loop diuretics are considered the first-line treatment for congestion, but their efficacy and safety in CRS are controversial, and resistance is common [25-27].

Several factors contribute to diuretic resistance; however, oral bioavailability is the first line of resistance in outpatient patients. Potential strategies to overcome diuretic resistance include increasing the dose and frequency, combining diuretic therapy, and changing the route of administration [28].

Diuresis can be optimized by administering diuretics via the subcutaneous route [29], since the bioavailability of diuretics is complete, whereas the oral route provides only 50% [28,29]. As such, it is essential that the nephrology nurse be able to administer medications via hypodermoclysis to their patients. This practice reduces the aggressiveness of treatment with diuretics. It contributes to the improvement of symptoms related to heart failure, and it is a safe alternative, with a low risk of complications [30,31].

However, the development of diuretic resistance often prompts the initiation of RRT, using various modalities, such as isolated ultrafiltration, hemofiltration, hemodialysis, and peritoneal dialysis, for the mechanical removal of fluids [28]. The nephrology nurse must be able to ensure the various dialysis techniques, which, although invasive, play a palliative role in symptom control and improving quality of life [22,32-35].

Among these techniques, peritoneal dialysis has shown superiority over hemodialysis in volume regulation and symptom control in the context of CRS, with improved quality of life, reduced hospitalization rates and duration, and increased patient survival [36]. In fact, studies suggest that extracorporeal methods invariably involve abrupt and aggressive changes in body volume and substantial circulatory stress, which can lead to frequent decompensations of CRS and the consequent need for hospitalization and loss of quality of life [22,32-36].

Given such a complex, progressive, and irreversible clinical condition, full of challenges in care management, it is essential that the nurse develop and implement a personalized follow-up plan for the rigorous monitoring and close follow-up of these patients. Furthermore, it is essential that the nurse promote health education sessions, strengthen self-confidence, empower the family in caregiving, conduct phone follow-up, and streamline coordination with primary healthcare and palliative care services. These patients also benefit from home care, especially the more fragile ones, reducing the need for visits to the hospital.

Nurse's role in conservative medical treatment for chronic kidney disease

The concept of providing palliative care for the terminal stage of CKD without resorting to dialysis, also known as conservative medical treatment, began to be introduced in 2003. It was suggested that dialysis might not always be in the best interest of patients, especially the elderly and highly dependent individuals. Today, it is a valid and universally established form of treatment [37].

Although dialysis can increase life expectancy and allow for a reasonable quality of life in selected elderly patients, most patients with severe comorbidities, frailty, functional impairment, or dementia tend to worsen with the initiation of dialysis. In fact, although the overall mortality rate of patients on hemodialysis is 12-14% [7], this percentage rises to 38% in patients over 75 years of age and can exceed 50% in elderly and frail patients [38].

This high mortality rate has raised questions about whether these elderly patients with significant comorbidities in the terminal stage of kidney disease have greater survival when treated with dialysis compared to conservative medical treatment [39]. Over the past few years, several studies have found little to no survival benefit or improvement in quality of life in patients on dialysis compared to what can be achieved through conservative medical treatment [40-42]. Furthermore, the evidence suggests that patients on conservative medical treatment spend less time hospitalized, undergo fewer invasive procedures, and die less frequently in a hospital setting compared to those who are dialyzed [43,44].

The discontinuation of dialysis in these patients is common and suggests that life on dialysis becomes unsustainable [45]. However, while patients who discontinue dialysis die within one to two weeks, those who refuse to start dialysis may live for months to years, with some studies indicating an average life expectancy of 6.3 to 23.4 months [46-48].

Thus, choosing dialysis should not be the default [45]. The decision between dialysis and conservative treatment in a frail patient with multiple comorbidities is complex, requiring a balanced and thoughtful approach, and should be shared with the patient and their family [45]. To this end, establishing the prognosis of these patients is essential to define a treatment plan tailored to their individual needs, avoiding unnecessary therapies and investigations [49].

It is important for the patient and their family to understand that, given the complexity of the clinical situation, non-dialytic management may be preferable, as it does not shorten the remaining lifespan but rather leads to a better quality of life [47]. The nurse's role in this decision involves using effective communication tailored to the patient and their family to help clarify relevant prognostic factors, including survival, symptom burden, functional trajectory, and quality of life with both dialysis and conservative treatment [45].

To this end, the nurse should begin by deconstructing common myths surrounding dialysis, such as the belief that it cures kidney failure, restores longevity, improves functional status, and eliminates the heavy symptom burden. It is also important to dispel the misconception that not starting dialysis means imminent death. While this may be true in life-threatening situations such as hyperkalemia or volume overload, the decision not to initiate chronic dialysis due to the progression of CKD to the terminal stage is compatible with life for several months, supported by residual kidney function [45].

Dealing with a highly limiting illness such as kidney disease is challenging and filled with emotions and uncertainties. In contact with the patient and their family, it is crucial for the nurse to develop a policy of non-abandonment, showing availability and providing easy access to healthcare services, as well as acting as a link to the rest of the conservative medical treatment team. The nurse should also empower the family in providing care, conduct follow-up phone calls, and facilitate coordination with primary healthcare and palliative care when necessary. Only in this way is it possible to provide personalized palliative care support, with the aim of optimizing the physical, emotional, and spiritual well-being of the patient and their family during the remaining time of their life.

In recent years, there has been significant progress in palliative care for end-stage CKD, with a better understanding of the pathophysiology and symptom management and improved prognostic tools [38]. This management includes careful attention to the symptoms that arise with the natural progression of the disease, such as nausea, vomiting, and anorexia, as well as the functional losses resulting from the clinical condition itself. It also involves correcting fluid and electrolyte imbalances, such as volume overload, acidosis, hyperkalemia, and phosphorus-calcium metabolism issues, and treating anemia associated with CKD.

In addition to educating and supporting medication management, the nurse should monitor blood pressure and use tools such as bioimpedance to assess the patient's body composition. Some bioimpedance parameters, such as phase angle and metabolic equivalents, are associated with the progression of frailty and sarcopenia, disability, and poor outcomes for geriatric patients, and can predict patients at higher risk to be identified for early action [50,51].

Conservative medical management of kidney disease is far from a "lack of treatment" but rather an active approach encompassing the medical, psychosocial, and cultural domains [45]. The success of this consultation will always depend on the development and implementation of personalized follow-up and close monitoring for a holistic, proactive, and multidisciplinary approach.

Nurse's role in managing expectations

Nurses are particularly well-placed to closely accompany patients and their families as they navigate the challenges of CKD progression and declining functional abilities. The aim is to empower patients and families to develop realistic expectations, communicate openly and honestly, and adjust those expectations as needed to effectively manage their journey. Indeed, managing expectations for these patients presents a complex yet crucial challenge. Expectations exist in all facets of life, and their impact varies depending on what we anticipate in each situation, which differs from person to person. It's about finding the balance between our aspirations and our realistic possibilities [52].

Given the inherent uncertainty of disease progression, the patient and their family face different contexts that profoundly influence the course of terminal illness. Each patient's experience, as well as the timeframe in which this progression occurs, is crucial to the development of the entire process, impacting not only their adaptation to the disease but also the organization and actions of the multidisciplinary team [53].

As the disease progresses and the patient faces an increasing level of disability, early treatment planning becomes essential. This process should involve understanding, communication, and discussion between the patient, family, and the multidisciplinary team to clarify end-of-life care preferences and align expectations regarding the progression of CKD. In this context, advance directives, such as a living will, do-not-resuscitate orders, and the appointment of a legal representative, are part of this process and play a fundamental role. It is crucial that patients are involved and take an active role in their own health journey (empowerment) [52]. Thus, the nurse's support becomes a crucial pillar in mitigating the emotional and social impacts of the disease, both for the patient and their family [53].

## Conclusions

The number of elderly and frail patients reaching the terminal stage of CKD has increased, making it essential to consider not only the symptomatic burden but also the significant impact of kidney replacement treatments on quality of life. The role of the nurse in the field of nephrology should go beyond the more invasive techniques of dialysis, integrating care that places the comfort and well-being of patients and their families at the center, as they face difficult decisions and experience the repercussions that the disease has on their quality of life and morbidity-mortality.
